# What Is the Optimal Value of the g-Ratio for Myelinated Fibers in the Rat CNS? *A Theoretical Approach*

**DOI:** 10.1371/journal.pone.0007754

**Published:** 2009-11-13

**Authors:** Taylor Chomiak, Bin Hu

**Affiliations:** Department of Clinical Neurosciences and Hotchkiss Brain Institute, University of Calgary, Calgary, Alberta, Canada; The Mental Health Research Institute of Victoria, Australia

## Abstract

**Background:**

The biological process underlying axonal myelination is complex and often prone to injury and disease. The ratio of the inner axonal diameter to the total outer diameter or g-ratio is widely utilized as a functional and structural index of optimal axonal myelination. Based on the speed of fiber conduction, Rushton was the first to derive a theoretical estimate of the optimal g-ratio of 0.6 [1]. This theoretical limit nicely explains the experimental data for myelinated axons obtained for some peripheral fibers but appears significantly lower than that found for CNS fibers. This is, however, hardly surprising given that in the CNS, axonal myelination must achieve multiple goals including reducing conduction delays, promoting conduction fidelity, lowering energy costs, and saving space.

**Methodology/Principal Findings:**

In this study we explore the notion that a balanced set-point can be achieved at a functional level as the micro-structure of individual axons becomes optimized, particularly for the central system where axons tend to be smaller and their myelin sheath thinner. We used an intuitive yet novel theoretical approach based on the fundamental biophysical properties describing axonal structure and function to show that an optimal g-ratio can be defined for the central nervous system (≈0.77). Furthermore, by reducing the influence of volume constraints on structural design by about 40%, this approach can also predict the g-ratio observed in some peripheral fibers (≈0.6).

**Conclusions/Significance:**

These results support the notion of optimization theory in nervous system design and construction and may also help explain why the central and peripheral systems have evolved different g-ratios as a result of volume constraints.

## Introduction

Myelination is a unique cellular process that can have a dramatic impact on the structure and physiology of an axon and its tissue surroundings. For example, as more myelin is added to an axon, the sheath gets thicker and begins to occupy an increasing proportion of the surrounding wire volume, altering the biophysical properties that describe axonal function. It is a widely held view that the g-ratio (the ratio of the inner axonal diameter to the total outer diameter) is a highly reliable ratio for assessing axonal myelination. Furthermore, it is generally believed that the g-ratio of a myelinated axon is optimized to achieve maximal efficiency and physiological optimization. This concept is supported by the observations that during the recovery process from demyelinating disease, central axons undergo an initial period of hyper-remyelination and increased diameters which then eventually revert to the normal g-ratio [2]–[5].

Rushton was the first to derive an optimal theoretical g-ratio of 0.6 [1]. In this classic study the calculation of g-ratio is based on the speed of fiber conduction. However, it was also realized that other aspects of axonal myelination including space and energy consumption likely influence the g-ratio [1], although this has not yet been accounted for. For example, in addition to Ruston's approach, subsequent models have also inherently neglected the influence of volume constraints on optimized design by holding the external diameter constant (i.e. fixed volume) [1], [6], [7]. Furthermore, Rushton's model was also later questioned with respect to the CNS where axons tend to be smaller and their myelin sheath thinner [8]. Here it was pointed out that conduction velocity maximization need not be the only criterion for optimized design, particularly for the CNS [8]. Indeed, if the speed of conduction and the minimization of conduction delays was the only concern regarding a given axons myelo-architectural design, then the g-ratio should be <0.25, since conduction speed continues to increase monotonically with increasing myelination [9]. Obviously, this is not supported by the experimental data especially on central myelinated fibers where g-ratio is significantly higher than 0.6 [10]–[16].

Few studies have thus far attempted to address the discrepancy regarding the g-ratio in CNS fibers estimated by these models and experimentally measured values. Conceptually, this discrepancy can be explained by the fact that these models are largely concerned with a single parameter (i.e. minimizing conduction delays) rather than the idea of system optimization. System optimization denotes a process through which an optimal solution naturally emerges from a set of alternatives to maximize favorable and minimize unfavorable outcomes [17]–[22]. This conjecture is intuitive in the case of axonal myelination if one considers that in order for the central system to compensate for continual increases in the total diameter of one axon, then the number and/or size and/or myelin thickness of the axons in the same wiring space will have to be reduced. Thus, a theoretical limit must be set for individual axons that does not allow for the unlimited expansion of the axons wire volume to outweigh the benefits associated with myelinating that axon. This theoretical limit, commonly defined as a global optimum, can be examined by evaluating the relationship between the biophysical properties describing axonal structure and function with increasing myelination.

## Results

### Theoretical Approach

**Figure 1:**
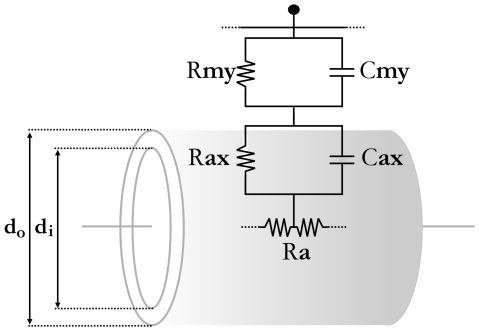
Geometrical and electrical properties of a myelinated axon internodal segment of unit length and a schematic of the equivalent circuit. *d_i_* and *d_o_* represent the inner and outer (i.e., *d_i_* + total myelin sheath thickness) axon diameters respectively. *R_m_* and *C_m_* used in the model can be related to the axolemma (ax) and myelin (my) electrical components as follows: *R_m_* = R_ax_+nR_my_ and 1/*C_m_* = 1/C_ax_+n/C_my_, where n is the number of myelin lamellae with a periodicity of 16 nm (naïve). R_a_ depends on both the geometric properties of the inner core and the core resistivity (*p*). Axon electrical parameters; *p* = 70 Ω·cm, R_ax_ = 4.7×10^3^ Ω·cm^2^, R_my_ = 800 Ω·cm^2^ (per lamellae), C_ax_ = 1 µF/cm^2^, C_my_ = 0.6 µF/cm^2^ (per lamellae). All values are based on published work (see [30], [59]–[62] and text).

When an axon internodal segment is progressively wrapped by myelin lamellae (Figure 1), the basic biophysical properties that are used to describe that axons structure-function relationship also change. With this, we can define that the efficiency of axon internodal myelination (*E_m_*) is proportional to the gains provided by the conservation of energy (*f*(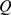)), minimization of conduction delays, i.e. via faster internodal membrane charging times resulting from reduced capacitance (*f*(*τ*)), and the preservation of conduction fidelity, i.e. via increased insulation and less transverse conductive leak (*f*(*λ*)). With the obvious need to conserve space in the CNS [23], an increasing wire volume resulting from an increasing sheath thickness works against the gains associated with increasing myelination and, as a result, wire volume (*f*(*v*)) is inversely related to *E_m_*: 
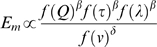
(1)where *f*(*x*) equals the property function (see below) and *β* and *δ* represent weighting factors. 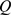, *τ* and *λ* are all based on the same electrical properties and since changing the weighting factor is equivalent to changing the electrical parameters, the weighting factor must be the same for each (i.e., *β*). *v*, on the other hand, does not depend on axonal electrical parameters and therefore can be differentially weighted (i.e., *δ*). Therefore, a balance between the gains and cost associated with increasing myelination must be negotiated for optimized myelo-architectural design.

Considering an axonal internodal segment of unit length (Figure 1), the function of the internodal segment is to first insulate and preserve conduction fidelity via reducing transverse conductive leak [24]–[26]. This feature can be related to the biophysical properties of the axon by *λ* (equation 2). As an internodal segment becomes more and more myelinated, *λ* increases, further reducing transverse conductive leak and increasing the spread of current [24], [25].
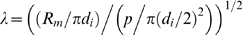
(2)where *R_m_* and *p* represent the specific membrane resistance (Ω·cm^2^) and axon core resistivity (Ω·cm) respectively. Increasing myelination reduces the segmental charging time (*τ*) by nonlinear decreases in capacitance per unit length (i.e., distance). This decrease in capacitance outweighs the increase in resistance, thus resulting in a net increase in the rate of internodal conduction [24], [25], [27], [28]. Furthermore, increasing myelination also reduces the charge quantity (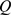), where 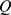 directly reflects the energy cost of conducting an action potential [27], [29]. These parameters can also be related to the biophysical properties of the axon by *τ* (equation 3) and 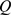 (equation 4) respectively,
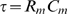
(3)

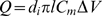
(4)where *l* and *C_m_* represent the unit length (cm) and the specific membrane capacitance (µF/cm^2^) respectively. An increasing wire volume (equation 5) resulting from an increasing sheath thickness works against the gains associated with increasing myelination.
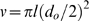
(5)


### General Relationship and Optima Function

**Figure 2:**
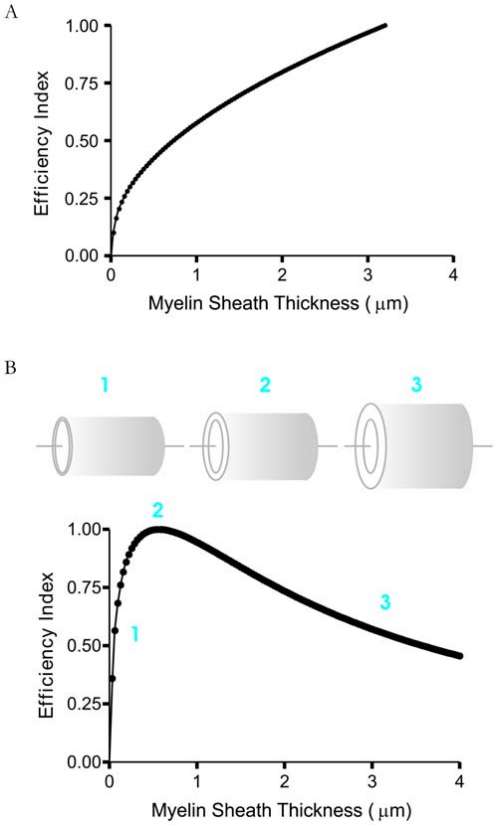
The model can predict an optimized level of myelination for a given axon. A: Relative efficiency index with increasing lamellae (i.e. increasing sheath thickness) without volume as a constraint (i.e., *δ* = 0). A global optimum does not exist. B: Top, a schematic illustrating a 2 µm inner diameter (*d_i_*) axon with an increasing (1→3) myelin sheath thickness; where the total myelin sheath thickness equals the difference between the outer (*d_o_*) and inner diameters (i.e., *d_o_*-*d_i_*). Bottom, relative efficiency index for different myelin sheath thicknesses. “2” represents the level of “optimized” (i.e, maximal efficiency) myelination for this particular axon. “1” and “3” illustrate that lower or higher levels of myelination provide a less efficient myelo-architectural design. Here volume is a constraint (*δ* = *β* = 1).

Since 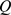 and *τ* decrease with axonal myelination, the relative efficiency gains (i.e., energy and charging time savings relative to that same internodal segment lacking myelin) can be expressed as 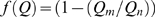 and 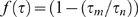 respectively; where *m* and *n* represent the myelinated and non-myelinated internodal segments respectively. *λ*, on the other hand, increases with axonal myelination and therefore the relative efficiency gains are expressed as 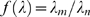. *v* also increases with axonal myelination and thus 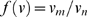. However, since space in the brain is severely limited, *v* works against design efficiency counteracting the gains resulting from axonal myelination. Hence, it can be considered as an “efficiency penalty”. The general relationship can therefore be expressed as shown in equation (1). We can define a new term, the Relative Efficiency Index (*E_i_*), to simplify things where;
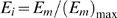



Therefore, with *E_m_*<(*E_m_*)_max_ and thus *E_i_*<1 (see Figure 2B), the global maximum and optimized myelo-architectural design will have a value of *E_i_* = 1 (i.e., *E_i_* = 1 = (*E_m_*)_max_/(*E_m_*)_max_).

### Nervous System g-Ratio Optimization

To evaluate the structural parameters in relation to system optimization, we first modeled an axon of 2 µm inner diameter (*d_i_*) and plotted the efficiency index with increasing myelin sheath thickness assuming that wire volume does not contribute to design optimization at all (i.e. *δ* = 0). Much like that reported by Smith and Koles based on conduction velocity alone [9], no optimized structure exists since efficiency continues to increase monotonically (Figure 2A). When however, wire volume is included in the model and it plays an equivalent role in optimizing design (i.e. *δ* = *β* = 1), an identifiable maximum or peak (marked as “2” in Figure 2B) is observed corresponding to a total myelin sheath thickness of 0.58 µm (0.29 µm on each side of axon, see Figure 1). This indicates that for this axon to utilize space efficiently while preserving spike fidelity and maximizing both spike conduction and energy savings, the fiber diameter (*d_o_* = *d_i_*+ total sheath thickness) must approach 2.58 µm. Having less myelin than this fails to maximize the potential gains from myelination (marked as “1” in Figure 2B), while having more than this, occupies too much volume and outweighs the gains associated with increasing myelination (marked as “3” in Figure 2B). This theoretical evaluation suggested that axon myelo-architectural design is represented in the biophysical properties of the axon itself, indicating that different caliber axons should have different optimized myelin sheath thicknesses.

**Figure 3:**
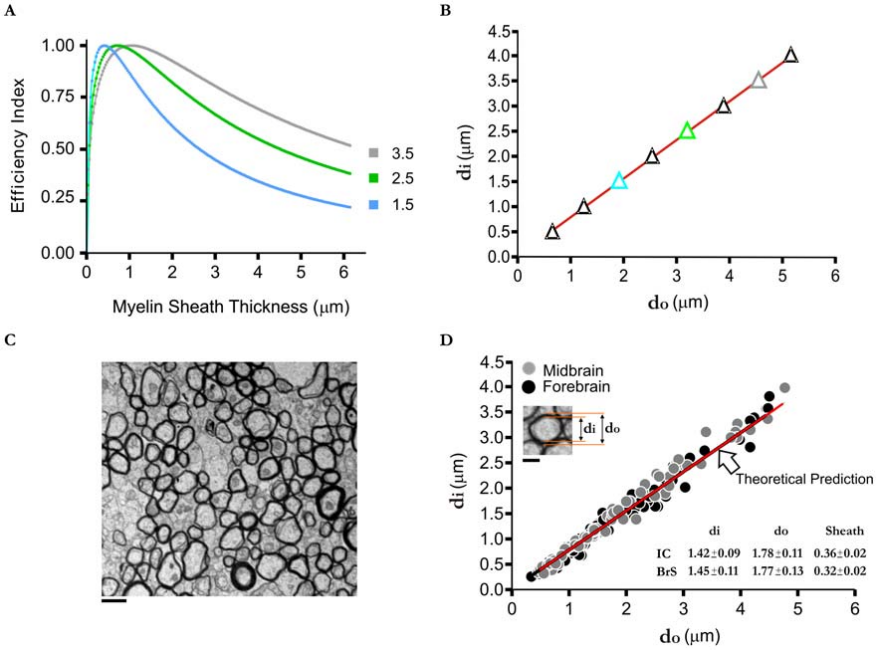
Model predictions for optimized axon myelo-architectural design (i.e., g-ratio) for different axon calibers of central white matter. A: Example relative efficiency index curves for increasing diameter axons (1.5-blue, 2.5-green and 3.5-grey) illustrating that the optimized level is scaled to axon inner diameter as indicated by the shifting of the peak or theoretical global maxima. For clarity, only 1.5, 2.5 and 3.5 µm caliber axon efficiency curves are shown. B: The theoretical predicted relationship between the inner (*d_i_*) and outer (*d_o_*) axon diameter for optimized axon myelo-architecture of increasing caliber when volume is a constraint (*δ* = *β* = 1). The model predicts that d_o_ and d_i_ are significantly correlated (correlation coefficient r = 1.0, R^2^ = 0.99; p<0.0001), where *d_i_* = (0.76–0.77)*d_o_*. *d_o_* = *d_i_* + total sheath thickness. Blue, green and grey triangles correspond to their respective curve in panel a. C: a representative TEM image illustrating a relatively thin myelin sheath thickness for most CNS axons. Scale bar  = 1 µm. D: Experimentally determined axon myelo-architecture for different axon calibers from rat brain. The experimentally determined relationship between the inner and outer axon diameters from the rat brain. Values are corrected for tissue shrinkage resulting from the fixation process (see Methods) to compare to the model prediction for naïve axons (Figure 3B). The red line represents the theoretically predicted relationship for optimized axon myelo-architectural design (model fit to experimental data: R^2^>0.96 for each). Inset: a typical example of a brain white matter axon indicating both d_i_ and d_o_. Midbrain: myelinated axons from the rat brainstem; forebrain: myelinated axons from the rat internal capsule. Summarized experimental values are listed for *d_i_*, *d_o_* and the sheath thickness (all in µm units). Inset scale bar  = 0.5 µm.

To examine whether this “optimized” myelo-architectural design can also apply to other axon calibers, we solved for the optimized myelin sheath thickness corresponding to the typical range (0.5–4.0 µm) of inner diameters observed in central white matter [12]. The model predicts that if axonal myelo-architecture is indeed a result of an optimized design, then there should be a tight linear correlation between the inner and outer diameters of these myelinated axons (i.e., a fixed g-ratio). To illustrate this, we first plotted the efficiency index curves for increasing calibers of axons. As shown in Figure 3A, the peak, or global maximum, is shifted to the right with increasing caliber of axon (only three have been shown for clarity). Next we plotted *d_i_* versus *d_o_* at maximal efficiency (i.e. corresponding the peak of the efficiency curve) for different axon calibers to determine the expected “optimized” g-ratio (Figure 3B). Here, the slope of the plot represents the g-ratio of axons as defined by the inner diameter of the axon (*d_i_*) divided by fiber diameter (*d_o_*). Indeed, our model reveals that system optimization of individual myelinated axons is achieved when the inner diameter approaches 77% of the outer diameter, i.e. a g-ratio of 0.76–0.77 (Figure 3A–D; theoretical model in panel B: r = 1.0, R^2^ = 0.99, p<0.0001; model fit to experimental data in panel D: R^2^>0.96 for each). Note, however, that g-ratio values are generally observed following histological processing and thus the expected experimentally observed g-ratio range at optimum efficiency would be on the order of 0.76 to just over 0.80 (g-ratio*_observed_* ≈0.76–0.81).

**Figure 4:**
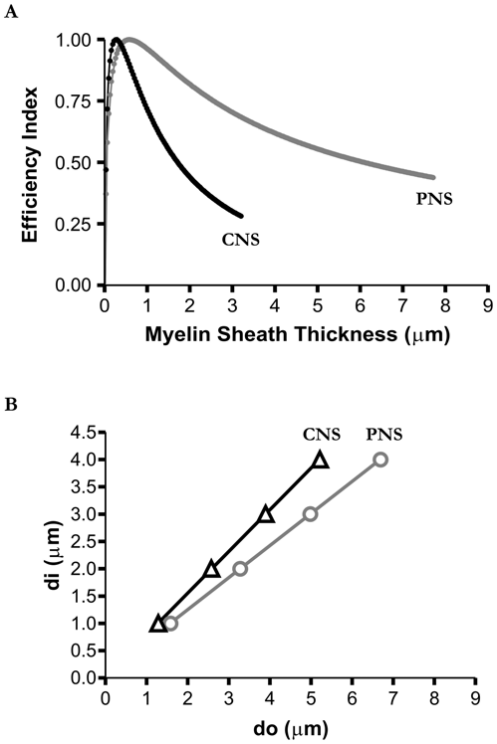
Optimized g-ratio for different neural systems. A: Efficiency index curves for a 1 µm diameter axon where δ = β = 1 (CNS-black curve) and where volume is less of a constraint *δ* = 0.6β (PNS-grey curve). Note that the peak is shifted to the right indicating that when volume is less of a constraint then the optimal sheath thickness is larger. B: Plots of *d_i_* versus *d_o_* representing different neural systems. In grey (circles) is when volume is less of a constraint (*δ* = 0.6*β*). In black (triangles) is when volume is as equally important as the other parameters (*δ* = *β* = 1) in defining an optimal structure (re-plotted from previous figure for comparison). The slopes of the lines, which represent the g-ratio, correspond to approximately 0.58–0.59 for the grey (PNS; correlation coefficient r = 1.0, R^2^ = 0.99; p<0.0001) and 0.76–0.77 for the black (CNS). Curves were generated using the parameters defined in Figure 1 and were the same for both the “CNS” and “PNS” plots with the exception of *δ*.

Finally, if the volume does not have as much of an influence on optimized axon myelo-architecture design, as may be predicted for some fibers in the PNS with thicker myelin sheaths, then a *δ*<*β* should be able to predict these lower PNS g-ratio values. To examine this, we reduced the influence of the volume constraint by roughly half to see if a thicker myelin sheath would represent the new optimized condition. Indeed, we found that reducing *δ* by about 40% (Figure 4A and B) resulted in optimized g-ratio values approaching that of some experimentally observed PNS fibers (e.g. 0.6) (see Table 1).

**Table 1:** Some summarized experimental g-ratio data.

CNS	g-Ratio	Source
**Corpus Callosum**	0.75–0.81	a–d
**Spinal Cord**	0.79	b
**Optic Nerve**	0.81	b, e–f
**Superior Cerebellar Peduncle**	0.76–0.81	g
**Anterior Commissure**	0.72–0.79	h
**Internal Capsule**	0.78^†^	
**Brainstem**	0.81^†^	
**PNS**		
**Sciatic**	0.55–0.68	f, i–k
**Sural**	0.47–0.6	l–m
**Saphenous**	0.61	k
**Hypoglossal**	0.69	n
**Facial**	0.69	n
**Splanchnic**	0.78	n
**Vagal**	0.73	n
**Glossopharyngeal**	0.78	n
**Oculomotor**	0.8	n
**Tibial**	0.69–0.76	o
**Trochlear**	0.71	p
**Phrenic**	0.54–0.59	q

Some previously published g-ratio values for myelinated axons. The data are reported as the mean value (or range of means - except for the anterior commissure that only reported a range). Note that mean values are in good agreement with our predictions (g-ratio*_observed_*≈0.76–0.81) for CNS and some PNS axons. Sources: a, [10]; b, [12]; c, [16]; d, [32]; e, [15]; f, [36]; g, [13]; h, [63]; i, [64]; j, [65]; k, [66]; l, [67]; m, [68]; n, [69]; o, [70]; p, [58]; q, [71]. ^†^signifies data from the present study (rat internal capsule raw data; 0.78±0.01 SEM, n = 85; and rat brainstem raw data; 0.81±0.01 SEM, n = 70).

## Discussion

In the present study we have developed a simple model describing how a myelinated axon can achieve optimal design by attaining a specific set of structural and functional parameters. We found that when volume is considered as an important limitation to the system, it lowers the set-point for which optimization occurs and results in a theoretical g-ratio boundary value that closely resembles the experimentally observed values for most central axons (Table 1). By contrast, when volume is less of a constraint on the system, thicker myelin sheaths are most efficient with g-ratios approaching that observed in some peripheral fibers (see Table 1).

### Theoretical Considerations

There are two important questions related to the basic findings reported in this study. First, does our simplified high myelin-resistance axon model (Figure 1) represent the real situation? The answer to this question can be gained by first examining the accuracy of other related axonal parameters derived from the same model. For example, if our theoretical framework approximates the physiological situation, then we should be able to predict the internodal resistance and capacitance measurements for a stretch of axon with specified dimensions (i.e., ≈10.5 µm inner diameter and 1 mm in length) [30]. Indeed, our simplified axon model predicts an internodal resistance of 277 MΩ and a capacitance of 1.79pF, which are consistent with the range of experimentally determined values of 220–350 MΩ and 1.2–1.9pF for internodal resistance and capacitance respectively [30]. Interestingly, these parameters correspond to g-ratio values on the order of 0.78 [31]. Second, does our simplified axon model represent the central nervous system? Central axons, particularly those located within the white matter of the mammalian brain, often preclude direct electrical property measurements due to their small size. However, based on the similar conduction properties among peripheral and central axons of equivalent caliber [32] and their common dependency on the axon's electrical properties [25], the structural parameters we used to construct our model (Figure 1) do not seem inappropriate. Indeed, both our experimental data on the g-ratio and those reported in previous studies are in good agreement with our model predictions (Table 1).

What about the other axon model that has been described previously? Blight and Someya reported a low myelin-resistance axon model [33], [34]. In this model, a total resistance of 145 MΩ, instead of 220 MΩ, was used to represent the same stretch of axon as that described by Tasaki (see above). We evaluated the predicted “optimized” axon myelo-architecture of this low myelin-resistance axon model and found that the g-ratio was >0.90 (e.g., optimal total sheath thickness of 0.19 µm for a 2 µm axon), a value that is inconsistent with previously reported values (Table 1). In fact, g-ratios as high as this are typically only seen under non-physiological conditions [10], [13] and approaches the theoretical value for spike failure [9]. Hence, the low myelin-resistance model may partially reflect current shunting (resulting from the puncture wound during microelectrode penetration [33], [34]) which can significantly distort and underestimate resistance measurements [35]. These data also suggests that our model possesses a high degree of sensitivity in estimating structural parameters, although it is built based on the simple notion of system optimization.

### Theoretical and Observed g-Ratios of Central and Peripheral Fibers

The experimentally measured g-ratios of central fibers (0.72–0.81; Table 1) seem to fall into a narrow range that is compatible with the expected theoretical estimate reported here (g-ratio*_observed_* ≈0.76–0.81). The typically <5% difference between the theoretical and observed data may be caused by certain uncontrollable factors in the latter as result of histological processing. However, for some peripheral axons, we do notice that the difference between our estimated g-ratio and that reported in the literature, which can approach 0.6 (see Table 1 for example), is significantly larger. It is known that some peripheral axon g-ratio values tend to be lower than central axon g-ratio values [36], although the underlying mechanism remains unclear. It is possible that in the peripheral system the space constraint is less of a limiting factor than in the brain, as axonal myelination in the PNS tends to be optimized for maximizing conduction velocity so that long projection axons can ensure rapid sensory and motor responses [1]. On the contrary, the relatively few but extremely large (e.g. >10 µm) diameter axons in some central systems may represent the case through which evolutionary pressure may have favored an even greater demand on the volume constraint. In other words, the volume constraint is likely more of an influence in much larger diameter axons since they inherently already occupy a larger volume, and thus, it may be predicted that much larger diameter axons as discussed by Paus and Toro [37] have slightly larger g-ratios that deviate from linearity.

### Functional Implications

Evolutionary optimization in brain systems was first attempted by Cajal about 100 years ago who mentioned the economy of space, time and matter as laws of brain maturation [38]. Some well-known examples of system optimization have since been described for the nervous system, including the neuropil wire fraction [39], and the adaptation of sparse coding and analog transmission through which energy savings associated with information transfer can be increased by distributed activity across networks of neurons and synapses [40], [41]. Optimization theory has also been applied to explain axonal network topology and distribution patterns [21], [23], [42], [43], neuronal morphology [44], and how grey/white matter volumes can be universally scaled across different species [45]. In these studies, it was shown that as the system operates optimally, a set of structural and functional parameters can emerge that define the physiological and/or structural boundaries. Our result is another example consistent with optimization theory and provides insight into the fundamental basis for central white matter design and construction.

Electrophysiological studies *in vitro* have shown that central axons are endowed with diverse conduction velocities and that regional axonal myelination can play an important role in influencing both the fidelity and timing of spike propagation [14], [46], [47]. These results are consistent with the notion that axons can utilize myelination as an adaptive mechanism to achieve rapid, reliable and energetically favorable information transmission. In this context, hyper-myelination and unrestricted spatial expansion of an axon is expected to incur significant cost to the system as a whole, since the other cellular elements occupying the same space and volume must modify their morphological properties in order to cope with the space “crunch” [39]. Thus, the g-ratio may be considered a reflection of the set-point at which the structural and functional organization of individual fibers has achieved a high degree of balance and optimization. This microscopic optimization of axonal myelo-architecture also supports the finding that fixed volume scaling can be seen between white matter and gray matter across different animal species [45], [48]. However, one question still remains: How is this set-point value and the globally optimized structure achieved? The underlying mechanisms may include not only the intrinsic electrical and biochemical properties of the axon itself [5], [49], but also active communication between axons and their local environment and neighboring glia [50]–[52].

## Methods

### Transmission Electron Microscopy

All experimental protocols were approved by the University of Calgary Conjoint Faculties Research Ethics Board (Protocol # MO8090). Under this protocol, animals are housed in The University of Calgary Animal Resource Center facility receiving constant care throughout the year. Tissue blocks of the forebrain and midbrain region of the mature rat brain were prepared as previously described [46]. Tissue blocks were immersed in 2.5% glutaraldehyde fixative/0.1 M sodium cacodylate buffer for one hour and then rinsed 0.1 M sodium cacodylate buffer three times ten minutes each. Tissue blocks were then post-fixed in 1% osmium tetroxide/0.1 M sodium cacodylate buffer for one hour, thoroughly rinsed three times five minutes each with distilled water, and then dehydrated with acetone. Tissues were embedded in 1∶1 acetone∶resin for one hour followed by 100% resin for one hour and then 100% resin overnight. Tissues were then placed in 100% resin for one hour before placing into moulds with fresh resin for polymerization in a 60°C oven overnight. Thin sections (0.07 µm) sections were collected on copper mesh grids for transmission electron microscopy. All specimens were examined under a Hitachi H-7000 electron microscope and images were acquired through a SIA-8 CCD camera mounted on the microscope for examination with Northern Eclipse Software (Cheektowaga, NY, USA). For morphological analysis, acquired images were analyzed for axon inner and outer diameters by taking the maximum and the minimum diameter and calculating the average as previously described [53].

### Tissue Shrinkage Correction

Although the absolute degree of tissue shrinkage and the naïve myelin periodicity can slightly vary, the total myelin sheath can be estimated in the naïve state by assuming a relative reduction in myelin tissue periodicity resulting from histological processing. This can be approximated by the apparent 2–3 nm discrepancy between the myelin periodicity observed via X-ray diffraction (naïve axons) and that for electron microscopy (fixed axons) as a result of dehydration and embedding during histological processing [54]. Since the myelin hydration layer (water layer) periodicity in the naïve state roughly corresponds to 2.5 nm [54], then dehydration results in a reduction from roughly 16 nm to 13.5 nm [55]–[58]. Therefore, a lamellae periodicity of 16 nm and 13.5 nm for the naïve and fixed axon respectively were used. As a result, the fixed myelin sheath was multiplied by 16/13.5 to approximate the true ratio in the naïve state.
